# A long waiting time from diagnosis to treatment decreases the survival of non-small cell lung cancer patients with stage IA1: A retrospective study

**DOI:** 10.3389/fsurg.2022.987075

**Published:** 2022-09-07

**Authors:** Bin Liu, Jia-Yi Qian, Lei-Lei Wu, Jun-Quan Zeng, Shu-Quan Xu, Jin-Hua Yuan, Yong-Liang Zheng, Dong Xie, Xiaolu Chen, Hai-Hong Yu

**Affiliations:** ^1^ Department of Oncology, The Affiliated Hospital of Jinggangshan University, Ji’an, China; ^2^ Department of Thoracic Surgery, Shanghai Pulmonary Hospital, School of Medicine, Tongji University, Shanghai, China; ^3^ School of Medicine, Tongji University, Shanghai, China; ^4^ Department of Respiratory and Critical Care, The Affiliated People’s Hospital of Ningbo University, Ningbo, China; ^5^ School of Medicine, Jinggangshan University, Ji'an, China

**Keywords:** non-small cell lung cancer, stage IA1, delayed treatment, prognosis, surgery

## Abstract

**Objective:**

The prognostic effect of delayed treatment on stage IA1 non-small cell lung cancer (NSCLC) patients is still unclear. This study aimed to explore the association between the waiting time before treatment and the prognosis in stage IA1 NSCLC patients.

**Methods:**

Eligible patients diagnosed with pathological stage IA1 NSCLC were included in this study. The clinical endpoints were overall survival (OS) and cancer-specific survival (CSS). The Kaplan-Meier method, the Log-rank test, univariable, and multivariable Cox regression analyses were used in this study. Propensity score matching was used to reduce the bias of data distribution.

**Results:**

There were eligible 957 patients in the study. The length of waiting time before treatment stratified the survival in patients [<3 months vs. ≥3-months, unadjusted hazard ratio (HR) = 0.481, *P *= 0.007; <2 months vs. ≥2-months, unadjusted HR = 0.564, *P *= 0.006; <1 month vs. ≥1-month, unadjusted HR = 0.537, *P *= 0.001]. The 5-year CSS rates were 95.0% and 77.0% in patients of waiting time within 3 months and over 3 months, respectively. After adjusting for other confounders, the waiting time was identified as an independent prognostic factor.

**Conclusions:**

A long waiting time before treatment may decrease the survival of stage IA1 NSCLC patients. We propose that the waiting time for those patients preferably is less than one month and should not exceed two months.

## Introduction

Lung cancer still has the first mortality among all malignancies, although, its incidence rate had decreased from the first rank to the second rank worldwide (1). The overall survival (OS) of lung cancer has improved due to the development of therapeutic approaches, such as third-generation targeted therapy and anti-PD-1 therapy (2, 3). However, previous studies confirmed that many factors affecting the prognosis led to the low OS rate (4–7). The waiting time from diagnosis to treatment, as a predictive factor for survival before treatment, were studied in some research. However, the conclusions from those studies were inconsistent. Some research suggested that a long waiting time from diagnosis to treatment could decrease the survival outcomes (8, 9), though other studies concluded that delay treatment did not affect the prognosis of patients significantly (10, 11). Thus, there is always a need to investigate the prognostic effect of a long waiting time before treatment on non-small cell lung cancer (NSCLC) patients.

With the popularization of computed tomography and radiomics, the rate of early diagnosis in NSCLC has increased (12, 13). Besides, the percentage of stage I NSCLC diagnosed has increased over the years (14). However, the survival of stage I presented heterogeneity according to the different clinical-pathological features (15, 16). In addition, in the current Covid-19 pandemic, overburdened medical services lead to a long waiting time before treatment (17). Nevertheless, the survival effect of a long waiting time before treatment on NSCLC patients with stage IA1 is unclear. Therefore, this study aimed to explore the maximum waiting time before therapy and its impact on survival outcomes of stage IA1 NSCLC patients and to provide reference information in the clinical practice.

## Patients and methods

### Patients

**Figure 1:**
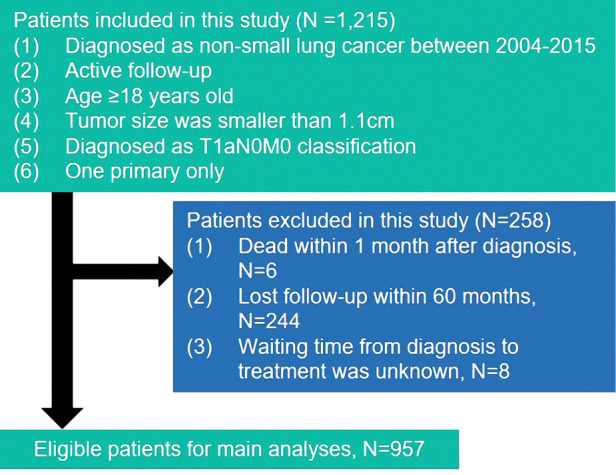
The flow chart of the present study.

This study includes all patients who were diagnosed with a histologically confirmed non-small cell during January 2004 to December 2015 from the Surveillance, Epidemiology, and End Results (SEER) database. Histology and site of disease were coded in SEER according to the International Classification of Diseases (ICD) for Oncology, Edition 3 (ICD-O-3). Patients who met the following criteria were enrolled in the study: (1) patients diagnosed as non-small cell lung cancer age ≥18 years old; (2) systemic assess tumor, node, and metastasis (TNM) staged at T1aN0M0 and pathologically confirmed tumor size was smaller than 1.1 cm; (3) patients had only one primary lesion. All patients were excluded if they were found the standard: (1) patients dead within 1 month after diagnosis; (2) patients lost follow-up within 60 months; (3) patients’ waiting time from diagnosis to treatment was unknow. The detailed information about selection standards are shown in Figure 1. Eventually, the study collected the information on 957 patients.

### Follow-up

The collected patients have a clear survival time and survival status. We regarded OS and cancer-specific survival (CSS) as observation endpoints. The CSS was from the date of diagnosis to the time of death caused by NSCLC, and the OS was from the date of diagnosis to the time of death. The survival time ranged from 1.0 to 154.0 months, with an average of 73.3 months.

### Statistical analysis

Statistical analysis was performed using SPSS Statistics 25.0 software (IBM SPSS, Inc., Chicago, IL, USA), R version 3.5.2 and GraphPad Prism 8. Univariable and multivariable Cox regression analyses were performed to calculate the hazard ratio (HR) and 95% confidence interval (CI) of the variables for overall survival and cancer-specific mortality. Those factors included sex, age, race, marital status, tumor location, surgical approach, chemotherapy, radiotherapy, grade, histological type and waiting time before treatment. A *P* value <0.05 was considered to be statistically significant. Survival curves were displayed using Kaplan-Meier curves and compared using the log-rank test. A multivariable proportional hazards model regression model was used to determine the association between treatment waiting time with other clinical features. Propensity score matching (PSM) was used to reduce the bias of data distribution. Statistical tests were based on a two-sided significance level.

## Results

### Patient characteristics

The clinical characteristics of patients in the study cohort are listed in Table 1. A total of 957 patients entered the analyses. Among these patients, females outnumbered males, constituting 64.8% of the patients. 436 (45.6%) patients were age 65 and below while 521 (54.4%) were over 65 years old. The majority of patients received surgical treatment, with limited resection accounting for 42.6% and lobectomy for 55.3%. Among the degree of tumor differentiation, there were 370 (38.7%) well differentiated, 294 (30.7%) of moderately differentiated, 177 (19.9%) of poor-undifferentiated, and a considerable part of tumor differentiation (*N* = 116, 12.1%) was unknown. In terms of tumor histological type, there were 564 (58.9%) patients with adenocarcinoma, 164 (17.1%) patients with squamous cell carcinoma, and 229 (23.9%) patients with unknown classification. Given the early stage of the tumor, more than 90% of patients undergo radiotherapy and only about 1.6% were treated with chemotherapy. More than half of patients waited less than 1 month to reach 56.7% (*N* = 543), and the proportion of patients who received surgery within 2 and 3 months were 79.4% and 91.6%, respectively.

**Table 1:** The characteristics of patients in this study.

Variables	Number of patients *N* = 957	Percent
Sex		
Male	337	35.2
Female	620	64.8
Age		
<66	436	45.6
>65	521	54.4
Histological type		
Adenocarcinoma	564	58.9
SCC	164	17.1
Other/unknown	229	23.9
Grade		
Well	370	38.7
Moderate	294	30.7
Poor-undifferentiated	177	18.5
Unknown	116	12.1
Surgical approach		
None	19	2.0
Limited resection	408	42.6
Lobectomy	529	55.3
Unknown	1	0.1
Chemotherapy		
None	942	98.4
Yes	15	1.6
Radiotherapy		
None	927	96.9
Yes	30	3.1
Race		
Caucasians	820	85.7
Other/unknown	137	14.3
Waiting time within 1 month		
No	414	43.3
Yes	543	56.7
Waiting time within 2 months		
No	197	20.6
Yes	760	79.4
Waiting time within 3 months		
No	80	8.4
Yes	877	91.6
Marital status		
None	391	40.9
Yes	533	55.7
Unknown	33	3.4
Laterality		
Left	363	37.9
Right	594	62.1
Location		
Upper	556	58.1
Lower	308	32.2
Other/unknown	93	9.7

SCC, squamous cell carcinoma.

### The independence test of propensity score matching dataset

We used PSM analysis to minimize the effect of waiting time within 3 months, whether the sex, age, histological type, tumor grade, surgical approach, to receive radiation or chemotherapy, race, marital status, laterality and location on OS or CSS. In the propensity scored-matched dataset, there were 78 pairs of patients, we found that *P* value of *χ*2 test or Fisher exact test after matching had no different from primary dataset (Supplementary Table S1).

### Univariable and multivariable analyses

Univariable Cox regression analysis was firstly performed to distinguish prognostic factors, and a total of 12 variables were included. The laterality and location of tumor distribution had no significant impact on overall mortality and cancer-specific mortality (Tables 2, 3). Excluding confounding factors, multivariable analysis confirmed sex, age, SCC, high grade, received surgery, radiotherapy, race and waiting time within 3 months were independent prognostic factors for OS (all *P* < 0.05, Table 2). Age, high grade, received surgery, chemotherapy, radiotherapy, race and waiting time within 3 months were independent prognostic factors for CSS (Table 3), a little bit distinguished from OS. Further subdivided the waiting time for surgery, and multivariable analysis showed waiting time within 2 months was independent prognostic factors for CSS (HR = 0.623, 95% CI, 0.409–0.949, *P* = 0.027, Table 4).

**Table 2:** The univariable and multivariable Cox regression analyses for patients’ overall mortality.

	Univariable analysis			Multivariable analysis		
	HR	95%Cl	*P*-value	HR	95%Cl	*P*-value
Sex						
Male	1	reference		1	reference	
Female	0.678	0.537–0.855	0.001	0.684	0.534–0.876	0.003
Age (years)						
<66	1	reference		1	reference	
>65	1.694	1.330–2.157	<0.001	1.486	1.157–1.909	0.002
Histological types						
Adenocarcinoma	1	reference		1	reference	
SCC	2.613	1.999–3.416	<0.001	1.546	1.152–2.075	0.004
Other/unknown	1.103	0.818–1.486	0.521	1.119	0.818–1.531	0.482
Grade						
Well	1	reference		1	reference	
Moderate	2.302	1.689–3.139	<0.001	1.880	1.352–2.614	<0.001
Poor-undifferentiated	3.340	2.421–4.607	<0.001	2.211	1.564–3.128	<0.001
Unknown	1.808	1.194–2.738	0.005	1.363	0.882–2.108	0.164
Surgical Approach						
None	1	reference		1	reference	
Limited resection	0.154	0.095–0.251	<0.001	0.411	0.204–0.830	0.013
Lobectomy	0.130	0.08–0.211	<0.001	0.354	0.174–0.723	0.004
Chemotherapy						
No	1	reference		1	reference	
Yes	3.452	1.836–6.492	<0.001	1.742	0.891–3.405	0.105
Radiotherapy						
No	1	reference		1	reference	
Yes	4.599	3.022–6.997	<0.001	2.167	1.183–3.968	0.012
Race						
Caucasians	1	reference		1	reference	
Other/unknown	0.528	0.353–0.790	0.002	0.566	0.375–0.852	0.0006
Waiting time within 3 months						
No	1	reference		1	reference	
Yes	0.597	0.419–0.851	0.004	0.632	0.441–0.906	0.013
Marital status						
None	1	reference		1	reference	
Married	0.723	0.571–0.914	0.007	0.833	0.649–1.069	0.150
Unknown	0.945	0.511–1.747	0.856	1.402	0.749–2.623	0.291
Laterality						
Left	1	reference				
Right	0.934	0.738–1.183	0.573			
Location						
Upper	1	reference				
Lower	0.899	0.697–1.159	0.410			
Other/unknown	0.642	0.408–1.009	0.055			

HR, hazard ratio; CI, confidence interval; SCC, squamous cell carcinoma.

Variable with *P*-value <0.05 in univariable analysis was incorporated in multivariable analysis.

The method of Cox regression was “Enter selection”.

**Table 3:** The univariable and multivariable Cox regression analyses for patients’ cancer-specific mortality.

	Univariable analysis			Multivariable analysis		
	HR	95%Cl	*P*-value	HR	95%Cl	*P*-value
Sex						
Male	1	reference		1	reference	
Female	0.808	0.549–1.189	0.280	0.873	0.586–1.300	0.503
Age (years)						
<66	1	reference		1	reference	
>65	1.828	1.226–2.725	0.003	1.565	1.036–2.364	0.033
Histological types						
Adenocarcinoma	1	reference		1	reference	
SCC	2.699	1.753–4.154	<0.001	1.366	0.860–2.171	0.187
Other/unknown	1.041	0.633–1.712	0.874	1.051	0.624–1.770	0.852
Grade						
Well	1	reference		1	reference	
Moderate	3.599	2.015–6.428	<0.001	3.126	1.714–5.701	<0.001
Poor-undifferentiated	5.779	3.213–10.39	<0.001	3.869	2.093–7.151	<0.001
Unknown	3.471	1.736–6.941	<0.001	2.391	1.153–4.958	0.019
Surgical Approach						
None	1	reference		1	reference	
Limited resection	0.093	0.048–0.183	<0.001	0.396	0.167–0.938	0.035
Lobectomy	0.084	0.044–0.163	<0.001	0.378	0.158–0.904	0.029
Chemotherapy						
No	1	reference		1	reference	
Yes	8.696	4.388–17.24	<0.001	4.101	1.953–8.611	<0.001
Radiotherapy						
No	1	reference		1	reference	
Yes	8.104	4.682–14.03	<0.001	3.170	1.537–6.538	0.002
Race						
Caucasians	1	reference		1	reference	
Other/unknown	0.368	0.171–0.792	0.011	0.413	0.191–0.893	0.025
Waiting time within 3 months						
No	1	reference		1	reference	
Yes	0.481	0.283–0.818	0.007	0.474	0.276–0.815	0.007
Marital status						
None	1	reference				
Married	0.760	0.518–1.114	0.159			
Unknown	0.945	0.341–2.615	0.913			
Laterality						
Left	1	reference				
Right	0.918	0.625–1.347	0.661			
Location						
Upper	1	reference				
Lower	1.088	0.731–1.618	0.678			
Other/unknown	0.425	0.171–1.054	0.065			

HR, hazard ratio; CI, confidence interval; SCC, squamous cell carcinoma.

Variable with *P*-value <0.05 in univariable analysis or sex was incorporated in multivariable analysis.

The method of Cox regression was “Enter selection”.

**Table 4:** Multivariable Cox regression for patients’ mortality based on different cut-off points of waiting time.

	Overall mortality			Cancer-specific mortality		
	HR	95%Cl	*P*-value	HR	95%Cl	*P*-value
Waiting time within 1 month						
No	1	reference		1	reference	
Yes	1.024	0.800–1.310	0.852	0.684	0.457–1.026	0.066
Waiting time within 2 months						
No	1	reference		1	reference	
Yes	0.792	0.604–1.039	0.092	0.623	0.409–0.949	0.027

HR, hazard ratio; CI, confidence interval; SCC, squamous cell carcinoma.

Variable with *P*-value <0.05 in univariable analysis, sex, or age was incorporated in multivariable analysis.

The method of Cox regression was “Enter selection”.

### Prognostic significance of treatment-waiting time

**Figure 2:**
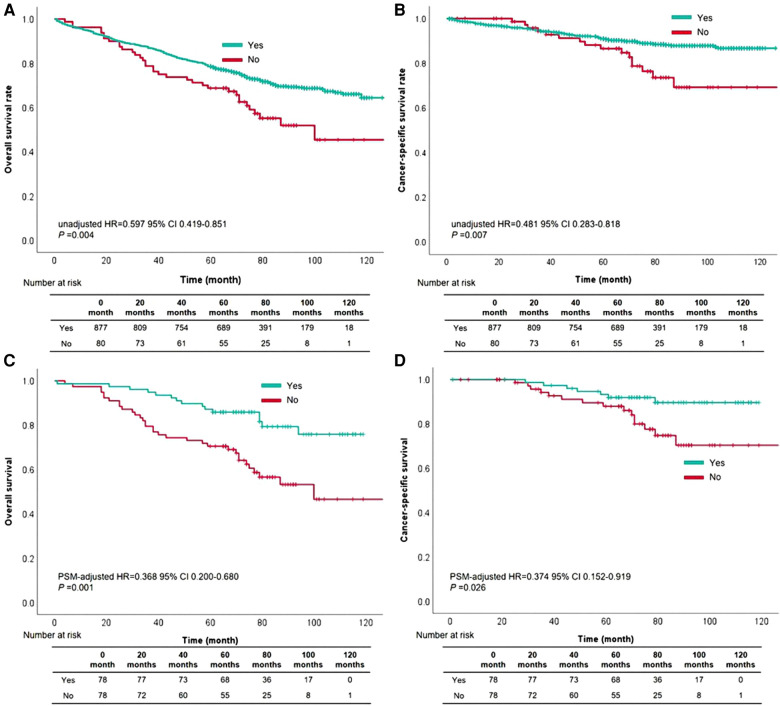
The survival curves based on waiting time within three months for overall survival (**A**) and cancer-specific survival (**B**) the survival curves based on waiting time within three months for overall survival (**C**) and cancer-specific survival (**D**) after propensity score matching.

Upon the results of univariable and multivariable analyses, waiting time for treatment within 3 months was a significant prognostic factor in this cohort. The 5-year OS rate was 63.0% and the 5-year CSS rate reached 85.0% in this cohort. The 5-year OS rates of patients with whose waiting time within 3 months or beyond 3 months were 65.0% and 48.0%, respectively (OS: unadjusted HR = 0.597, 95% CI, 0.419–0.851, *P* = 0.004, Figure 2A). For CSS rate stratified by treatment waiting time, those whose waiting time within 3 months had a better survival than those with waiting time beyond 3 months (unadjusted HR = 0.481, 95% CI, 0.282–0.818, *P* = 0.007, Figure 2B). Although factors such as age, sex, and tumor grade did not influence, we used 78 pairs of patients after PSM for survival analysis. After removing possible underlying factors, we could see patients with waiting time within 3 months had a better survival than those with waiting time over 3 months (OS: PSM-adjusted HR = 0.368, 95% CI, 0.200–0.680, *P* = 0.001, Figure 2C; CSS: PSM-adjusted HR = 0.374, 95% CI, 0.152–0.919, *P* = 0.026, Figure 2D).

**Figure 3:**
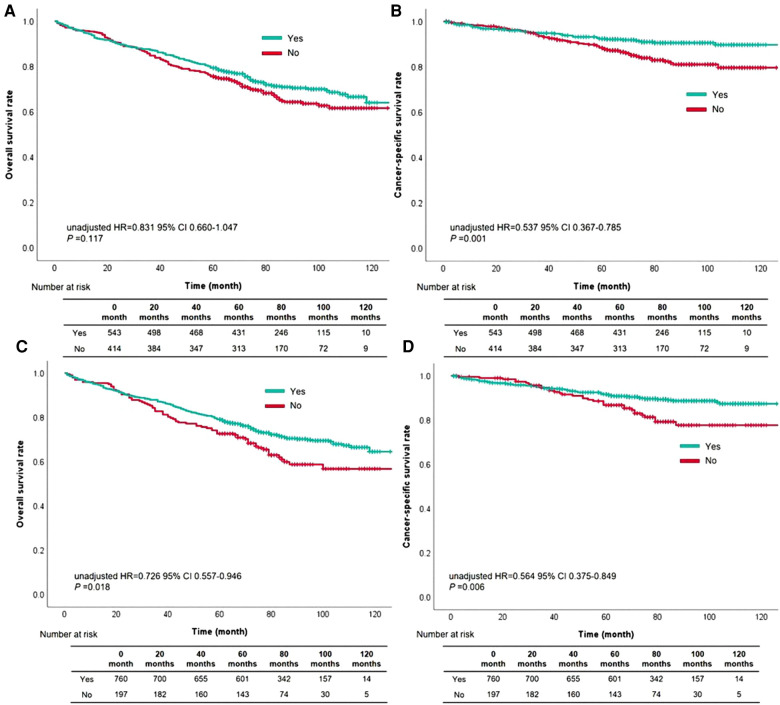
The survival curves based on waiting time within one month for overall survival (**A**) and cancer-specific survival (**B**), or waiting time within two months for overall survival (**C**) and cancer-specific survival (**D**).

The cutoff value of waiting time was further subdivided into within one month and two months, comparing the prognostic level was compared between the two groups. OS rate based on waiting time within one month compared with over one month had no difference (unadjusted HR = 0.831, 95% CI, 0.660–1.047, *P* = 0.117, Figure 3A). As for CSS rate, cases with waiting time within one month had more satisfactory outcomes than those with waiting time over one month (unadjusted HR = 0.537, 95% CI, 0.367–0.785, *P* = 0.001, Figure 3B). Besides, patients with waiting time within two months had a better survival than those with waiting time over two months (OS: unadjusted HR = 0.726, 95% CI, 0.557–0.946, *P* = 0.018, Figure 3C; CSS: unadjusted HR = 0.564, 95% CI, 0.375–0.849, *P* = 0.006, Figure 3D).

## Discussion

In this study, we used the data of 957 patients to investigate the prognostic impact of a long waiting time before treatment on stage IA1 NSCLC patients. The Kaplan-Meier method was used to compare the significance of different waiting times in the stage IA1 NSCLC patients. The results generated from the analysis of the Kaplan-Meier method showed that waiting time before treatment over one month decreased CSS rates in stage IA1 patients. However, the OS rates were only reduced when the waiting time was longer than two months for the same cohort. Furthermore, univariable and multivariable Cox regression analyses were conducted to identify the overall mortality and cancer-specific mortality. As previous studies presented, females accounted for a higher percentage of early-stage NSCLC than males (16, 18). Besides, the most part of histological type was adenocarcinoma in the small-sized NSCLC (16). Patients with adenocarcinoma had much better survival than cases with squamous cell carcinoma (16, 19). Our study showed the same results as those studies.

In this cohort with tumor size ≤1.0 cm, over 90% of patients received surgical resection. Therefore, the 5-year CSS rate reached 85%. We found that waiting time within one month, two months, or three months could improve the prognosis of NSCLC patients with stage IA1 after univariable analysis, although the prognosis of this cohort was satisfactory. After adjusting for other confounders, only patients could significantly get survival benefits whose waiting time was shorter than three months before treatment. Interestingly, the CSS rates could be increased when the waiting time was shorter than two months in the multivariable Cox regression model. Besides, the *P*-value of 0.066 was closed to 0.05 in the multivariable analysis when the cut-off point of waiting time was one month. These results might suggest that the waiting time from diagnosis to treatment mainly affected CSS of stage IA1 patients. Therefore, we propose that the waiting time for NSCLC patients with stage IA1 from diagnosis to treatment preferably is less than one month and should not exceed two months.

There is a long-standing discussion of the impact of waiting time from diagnosis to treatment on patient prognosis. The study results from *Diaconescu R et al.* showed that the shorter waiting time was associated with shorter survival time (20). However, they only analyzed the data of 495 patients in one cancer center. A recent report from *Klarenbeek SE et al.* based on a nationwide observational cohort study presented that a more rapid start of therapy did not improve survival outcomes in NSCLC patients with advanced stage (10). The abovementioned research mainly collected NSCLC patients with clinical stage III-IV, though its sample size was large. With the popularization of computed tomography and the development of radiomics, the rate of early diagnosis in NSCLC has increased (12, 13). Some researchers have paid attention to the study of the association between waiting time before therapy and patient survival. Two studies from The United States and South Korea confirmed that a long waiting time before treatment could have a survival effect on early-stage NSCLC patients (9, 21). Besides, delayed treatment was considered mainly affect the long-term mortality rather than short-term survival, such as 5-year mortality (9). However, the above two studies did not classify the detailed groups according to tumor size in cohort with stage I. In the present study, we focused on small-size NSCLC patients and analyzed the data of tumor size ≤1.0 cm. Based on the different observational endpoints (overall mortality and cancer-specific mortality), different cut-off values of waiting time had a different effect on the prognosis of stage IA1 NSCLC patients. Finally, we suggest that patients receive treatment early in clinical practice.

The effect of delayed treatment on the survival of NSCLC patients was inconsistent (8–10, 20, 21). There are two main reasons for this phenomenon. On the one hand, the cohort from different studies had different treatment approaches due to the different proportions of combined stages. Some patients received timely therapy; perhaps the emergency treatment was due to the urgency of the condition. Therefore, the poorer prognosis of such patients should be due to the condition itself and not to the length of waiting time before therapy (10). On the other hand, some patients did not receive complete essential checks, although those patients underwent timely treatment. However, the treatment received was not optimal for this patient before the detailed diagnosis. Regrettably, the studies mentioned above did not solve those problems. Thus, we still need a prospective study that may be able to explore these questions better.

With the pandemic of Covid-19, the carrying capacity of healthcare systems is being challenged worldwide (22, 23). It is difficult for some patients to receive therapy early (24). Previous studies confirmed that greater psychological distress was associated with slower time to treatment in oncological patients (25, 26). Besides, the psychological burden negatively influenced survival quality and prognosis in patients with malignant tumors (27, 28). Our findings showed that the waiting time ≥3 months had a significant negative effect on CSS and OS for stage IA1 NSCLC patients after adjusting for other confounders, and the waiting time ≥2 months only influenced OS rates. Although, in general, it is essential to start treatment as early as possible, the present study found that delayed treatment within a limited time might be acceptable. This finding is likely to relieve the patient's psychological burden and help the doctor to arrange the patient's treatment appropriately.

There are some drawbacks in the present study. First, the detailed information about waiting days was not obtain from SEER database; therefore, we only analyzed waiting time based on unit of month. Second, the radiological features (such as consolidation-to-tumor ratio) of small-sized tumor were unknown in the lung. Thus, further analysis of small nodules, such as the classification of ground glass nodules or solid nodules, cannot be performed. Third, the selection bias is inevitable, since this study belongs to a retrospective study. Fourth, the information on driver genes and pathological features (such as epidermal growth factor receptor and vascular-lymphatic invasion) was not detailed in the SEER database. Therefore, the detailed data from our hospital was needed to further analysis in the next study. Finally, we need reproducible studies to confirm our findings.

## Conclusions

A long waiting time from diagnosis to treatment may decrease the survival of stage IA1 NSCLC patients. We propose that the waiting time for NSCLC patients with stage IA1 before therapy preferably is less than one month and should not exceed two months.

## Data Availability

The raw data supporting the conclusions of this article will be made available by the authors, without undue reservation.
