# Supplementary material for: A long waiting time from diagnosis to treatment decreases the survival of non-small cell lung cancer patients with stage IA1: A retrospective study
Source: Front Surg. 2022 Sep 7;9:987075. doi: 10.3389/fsurg.2022.987075 (PMC9489994; doi:10.3389/fsurg.2022.987075)
Supplement: Supplementary file 1 [file Table_1_v2.docx]

**Supplementary table 1** The characteristics of patients in this study after propensity score matching.

| **Variables** | **Waiting time within 3 months** | | ***P*-value** |
| --- | --- | --- | --- |
|  | **No (n=78)** | **Yes (n=78)** |  |
| Sex |  |  | 0.742 |
| Male | 31 (39.7%) | 29 (37.2%) |  |
| Female | 47 (60.3%) | 49 (62.8%) |  |
| Age |  |  | 0.521 |
| <66 | 39 (50.0%) | 35 (44.9%) |  |
| >65 | 39 (50.0%) | 43 (55.1%) |  |
| Histological type |  |  | 0.925 |
| Adenocarcinoma | 45 (57.7%) | 43 (55.1%) |  |
| SCC | 17 (21.8%) | 19 (24.4%) |  |
| Other/ unknown | 16 (20.5%) | 16 (20.5%) |  |
| Grade |  |  | 0.561 |
| Well | 26 (33.3%) | 28 (35.9%) |  |
| Moderate | 23 (29.5%) | 29 (37.2%) |  |
| Poor-undifferentiated | 19 (24.4%) | 14 (17.9%) |  |
| Unknown | 10 (12.8%) | 7 (9.0%) |  |
| Surgical approach |  |  | 0.903* |
| None | 2 (2.6%) | 1 (1.3%) |  |
| Limited resection | 33 (42.3%) | 35 (44.9%) |  |
| Lobectomy | 43 (55.1%) | 42 (53.8%) |  |
| Chemotherapy |  |  | NA |
| None | 78 (100%) | 78 (100%) |  |
| Radiotherapy |  |  | 1.0* |
| None | 75 (96.2%) | 76 (97.4%) |  |
| Yes | 3 (3.8%) | 2 (2.6%) |  |
| Race |  |  | 0.104 |
| Caucasians | 67 (85.9%) | 59 (75.6%) |  |
| Other/ unknown | 11 (14.1%) | 19 (24.4%) |  |
| Marital status |  |  | 0.509* |
| None | 46 (59.0%) | 50 (64.1%) |  |
| Yes | 32 (41.0%) | 27 (34.6%) |  |
| Unknown | 0 (0.0%) | 1 (1.3%) |  |
| Laterality |  |  | 0.873 |
| Left | 39 (50.0%) | 38 (48.7%) |  |
| Right | 39 (50.0%) | 40 (51.3%) |  |
| Location |  |  | 0.480 |
| Upper | 42 (53.8%) | 35 (44.9%) |  |
| Lower | 31 (39.7%) | 35 (44.9%) |  |
| Other/ unknown | 5 (6.5%) | 8 (10.2%) |  |

NA: there was no *P*-value. *: *P*-value was calculated by Fisher exact test.
